# In vitro investigation of the blood flow downstream of a 3D-printed aortic valve

**DOI:** 10.1038/s41598-024-51676-6

**Published:** 2024-01-18

**Authors:** Till Zeugin, Fergal B. Coulter, Utku Gülan, André R. Studart, Markus Holzner

**Affiliations:** 1 Department of Civil, Environmental and Geomatic Engineering, Institute of Environmental Engineering, Swiss Federal Institute of Technology ETH Zürich, Zürich, Switzerland; 2 Swiss Federal Institute for Forest, Snow and Landscape Research WSL, Birmensdorf, Switzerland; 3 Complex Materials, Swiss Federal Institute of Technology ETH Zürich, Zürich, Switzerland; 4 Hi-D Imaging, Winterthur, Switzerland; 5 Swiss Federal Institute for Water Science and Technology EAWAG, Dübendorf, Switzerland; 6 Institute of Hydraulic Engineering and River Research (IWA), University of Natural Resources and Life Sciences, Vienna, Austria

**Keywords:** Biomedical engineering, Fluid dynamics

## Abstract

The hemodynamics in the aorta as well as the durability of aortic valve prostheses vary greatly between different types of devices. Although placement and sizing of surgical aortic valve prostheses are excellent, the valve geometry of common devices cannot be customized to fit the patient’s anatomy perfectly. Similarly, transcatheter aortic valve implantation (TAVI) devices are not customizable and may be orientated unfavorably during implantation. Imperfect fit of an aortic valve prosthesis may result in suboptimal performance and in some cases the need for additional surgery. Leveraging the advent of precision, multi-material 3D-printing, a bioinspired silicone aortic valve was developed. The manufacturing technique makes it fully customizable and significantly cheaper to develop and produce than common prostheses. In this study, we assess the hemodynamic performance of such a 3D-printed aortic valve and compare it to two TAVI devices as well as to a severely stenosed valve. We investigate the blood flow distal to the valve in an anatomically accurate, compliant aorta model via three-dimensional particle tracking velocimetry measurements. Our results demonstrate that the 3D-printed aortic valve induces flow patterns and topology compatible with the TAVI valves and showing similarity to healthy aortic blood flow. Compared to the stenosis, the 3D-printed aortic valve reduces turbulent kinetic energy levels and irreversible energy losses by over 75%, reaching values compatible with healthy subjects and conventional TAVIs. Our study substantiates that the 3D-printed heart valve displays a hemodynamic performance similar to established devices and underscores its potential for driving innovation towards patient specific valve prostheses.

## Introduction

Aortic stenosis, predominantly caused by calcification of the aortic valve leaflets, is a prevalent cardiovascular disease with high morbidity and mortality^1^. It occurs more frequently in older people, where roughly 3% of patients over 75 years of age are affected^2^. Aortic stenosis causes increased leaflet stiffness and a reduced aortic valve orifice area. This leads to an elevated transaortic pressure gradient and consequently a higher workload for the left ventricle^2^. Apart from the strain on the heart, malfunction of the aortic valve alters the aortic blood flow and can lead to increased turbulence and elevated shear stresses^3^. Friction at the aortic wall may also be altered, which can cause endothelial damage^4^. Left untreated, aortic stenosis mortality is approximately 75% within 3 years of the onset of symptoms^5^. Recommended treatment for symptomatic patients is aortic valve replacement. As society’s average age continues to rise, aortic valve replacements are in increasing demand. The annual number of patients needing aortic valve replacements is estimated to almost triple from ca. $290'000$ in 2003 to more than $850'000$ by the year 2050^6^.

The most common procedure is the implantation of an artificial valve during open-heart surgery, where the native valve is removed and replaced with either a mechanical or an artificial tissue valve. Mechanical valves are durable, however induce unphysiological hemodynamics including turbulence and high shear stresses on the blood cells which can lead to hemolysis and thrombi^7^. The latter requires life-long use of anticoagulation drugs as a countermeasure. Tissue valves exhibit significantly better hemodynamics but suffer from longevity issues mostly due to calcification of the leaflets leading to incomplete closure and leakage^8^. Furthermore, open-heart surgery poses additional risks and thus may not be suitable for patients with severe comorbidities^9^. An alternative solution that mitigates problems associated with an invasive open-heart surgery is based on the implantation of a prosthetic valve through the femoral artery termed transcatheter aortic valve implantation (TAVI). TAVI devices offer exceptional hemodynamic performance^9^ though inherently share the same durability issues as tissue valves. Complications of TAVIs include damage to the endothelium, caused by the valves metallic stent, leading to inflammation and hyperplasia^10^, as well as thrombosis^11^. An important drawback all current valve replacement methods share is non-patient-specific valve geometries lead to a suboptimal fit of the valve in the patient’s aorta, which may cause paravalvular leakage and altered blood flow topology. Moreover, artificial heart valves are expensive and highly time consuming to develop and produce.

Recently, a new technique to 3D-print tri-leaflet aortic valves in silicone was developed, providing fully customizable bio-inspired aortic valve phantoms and laying the foundations for patient-specific valve prostheses via additive manufacturing of suitable materials^12^. Polymeric valves promise a merging of the superior durability of mechanical valves and the more physiological hemodynamics of bioprosthetic valves^13^. In fact, initial testing of the 3D-printed aortic valve in a pulse duplicator under accelerated conditions showed both excellent longevity and performance in terms of valve dynamics and in preventing leakage.

Investigation of the aortic blood flow downstream of an aortic valve prosthesis allows for further assessment of the valve’s hemodynamic performance. In vivo and in vitro flow measurement techniques as well as Computational Fluid Dynamics have been applied to study aortic blood flow^14–19^. Commonly used metrics include phase averaged velocities, Reynolds stresses, mean and turbulent kinetic energy, shear stresses as well as helicity. Similar analysis has been carried out recently to characterize the hemodynamic performance of artificial aortic valves; Ge *et al.*^20^ studied Reynolds and viscous stresses near valve leaflets, whereas von Knobelsdorff-Brenkenhoff *et al.*^21^ quantified wall shear stresses and graded helicity as well as flow eccentricity in the ascending aorta distal to artificial aortic valves. In an experimental study, Gülan *et al.*^22^ assessed the mean and turbulent kinetic energy as well as shear stresses downstream of TAVI prostheses, while Corso *et al.*^23^ additionally used coherent helical and vortical structures as well as blood damage indices to quantify valve performance.

In this study, we investigate the hemodynamic performance of a newly developed 3D-printed aortic valve (3D-printed AV) and compare it to a severly stenosed case, a bioprosthetic TAVI valve and a polymeric TAVI device. To this end, we carry out in vitro three-dimensional particle tracking velocimetry (3D-PTV) measurements of the blood flow in an anatomically accurate, compliant aorta model under physiological conditions. We determine and compare retrograde flow, helical structures and helicity intensity, as well as mean and turbulent kinetic energy and dissipation rates to characterise the hemodynamic performance of the 3D-printed AV.

## Results

For the qualitative analysis of the hemodynamics, we investigate flow patterns, helical structures and the spatial distribution of mean and turbulent kinetic energies.

**Figure 1:**
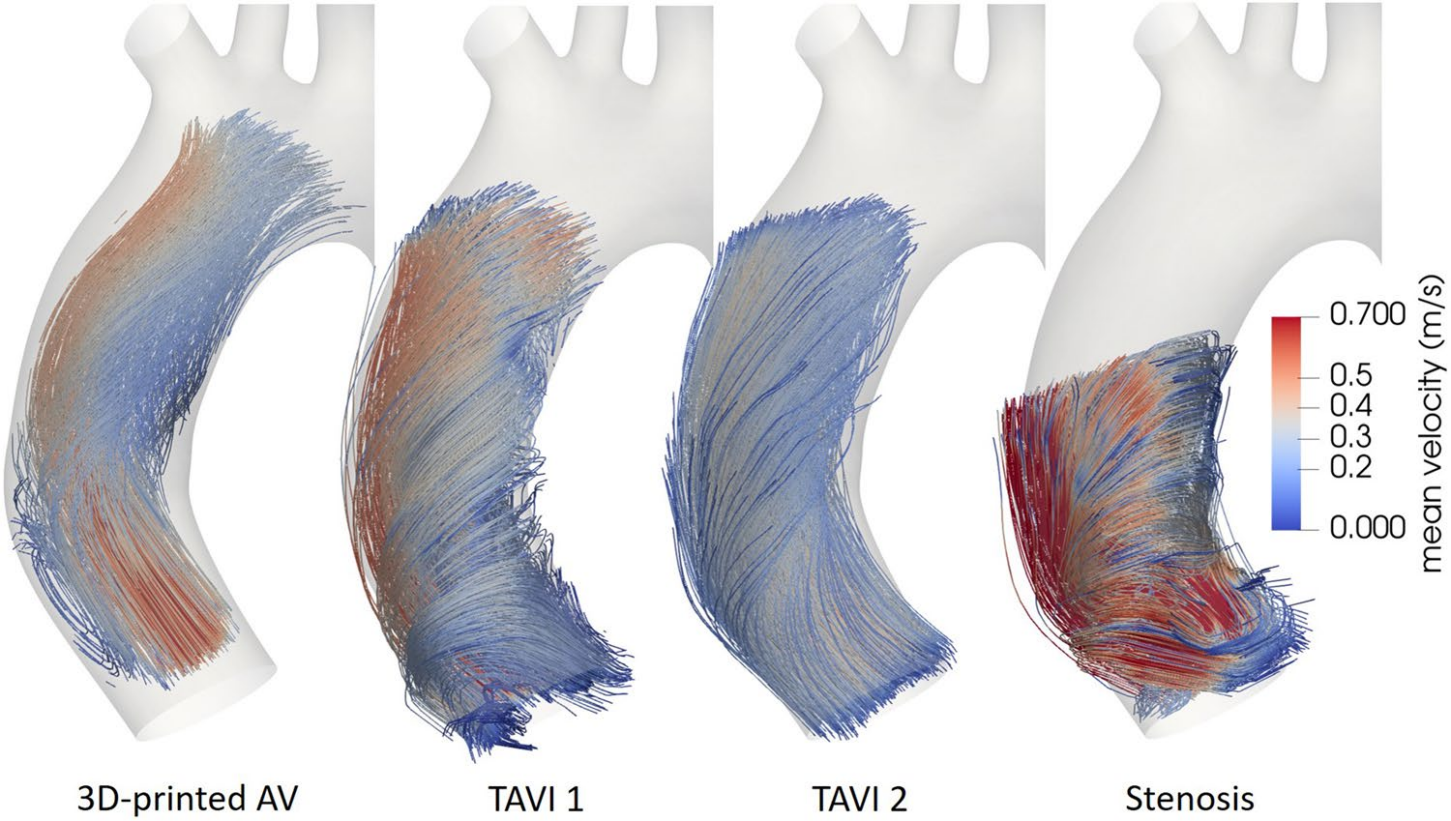
Streamlines at peak systole for the four studied cases color-coded with mean velocity magnitude.

Streamlines were generated on the basis of the phase-averaged Eulerian flow fields for the assessment of the flow patterns downstream of the valves. The origins of the streamlines were uniformly distributed in a sphere with a diameter equal to that of the aorta phantom inlet. The streamlines for all cases at peak systole are presented in Fig. 1 and exhibit a conspicuous right-handed rotation along the inner wall of the ascending aorta (AAo).

**Figure 2:**
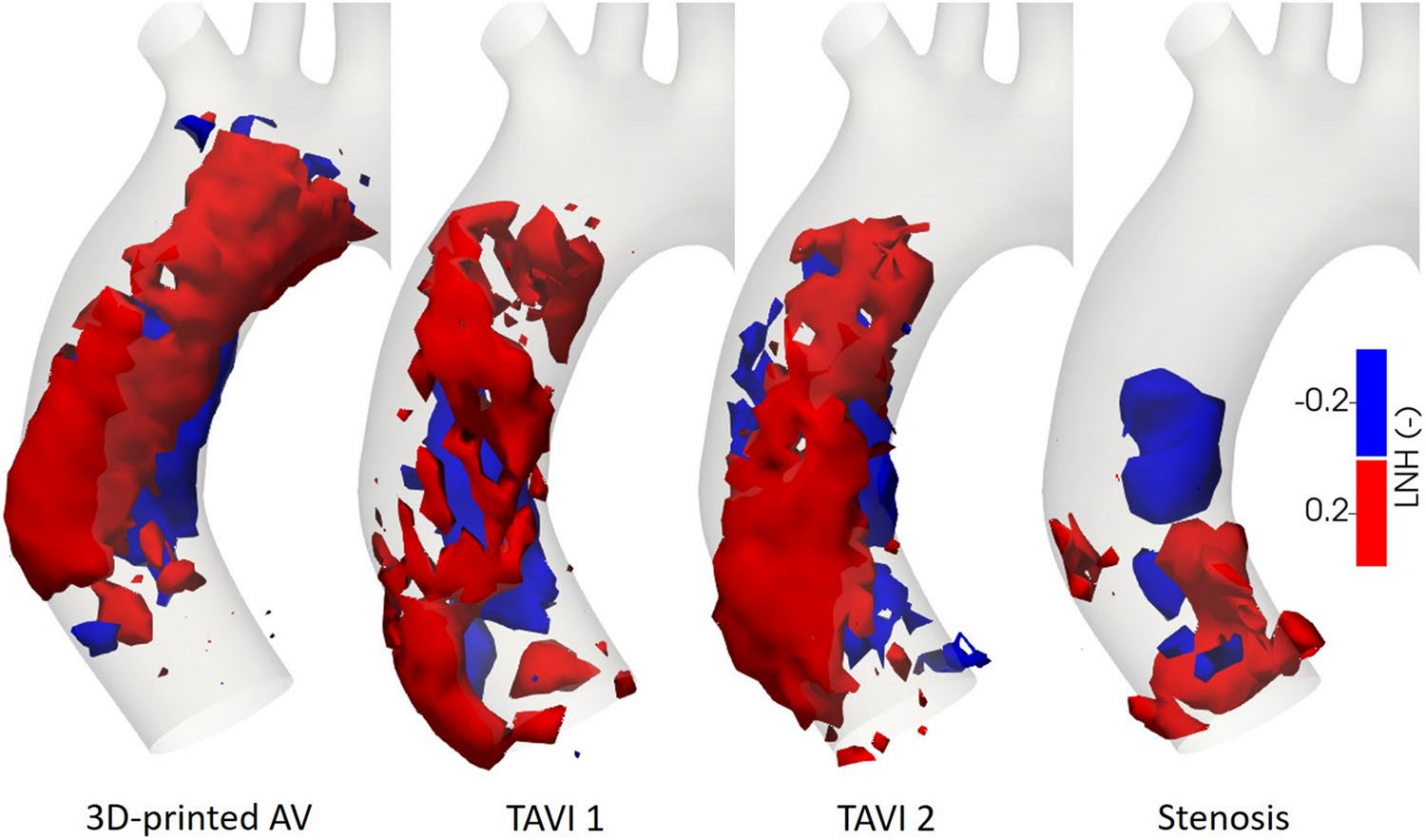
Pulse averaged local normalized helicity for the four different cases. Positive (negative) values indicate right-(left-)handed fluid structures.

Figure 2 shows positive and negative isosurfaces of the pulse averaged local normalized helicity (LNH), allowing for further assessment of the flow topology. Additionally to the mean topology, we investigate the evolution of helical structures over the duration of a pulse (see Supplementary Material, SM, Fig. S5). The temporal evolution of the helical structures follows the same basic sequence for the cases of the 3D-printed AV and TAVI 2, where helicity is present already during the accelerating phase though no larger helical structures have been formed yet. During the systolic peak, two counter-rotating helical structures form, where the one towards the inner wall is right-handed and the structure at the outer wall rotates left-handedly. This flow topology persists during the deceleration phase where rotational inertia dominates. Towards the end of the deceleration, the left-handed structure becomes dominant before the helical structures break down at the beginning of systole. For TAVI 1, coherent counter-rotating helical structures appear during the first part of systole and quickly break down approaching peak systole. During diastole the flow is characterized by a left-handed helical structure moving from the inner to the outer aortic wall. In the case of the severe stenosis, a large right-handed helical structure occurs during early systole. Approaching peak systole this structure breaks down and numerous small patches of both positive and negative LNH values are present, though no coherent structures are formed. Only during advanced diastole a coherent left-handed helical structure is formed which persists until the beginning of the new pulse.

**Figure 3:**
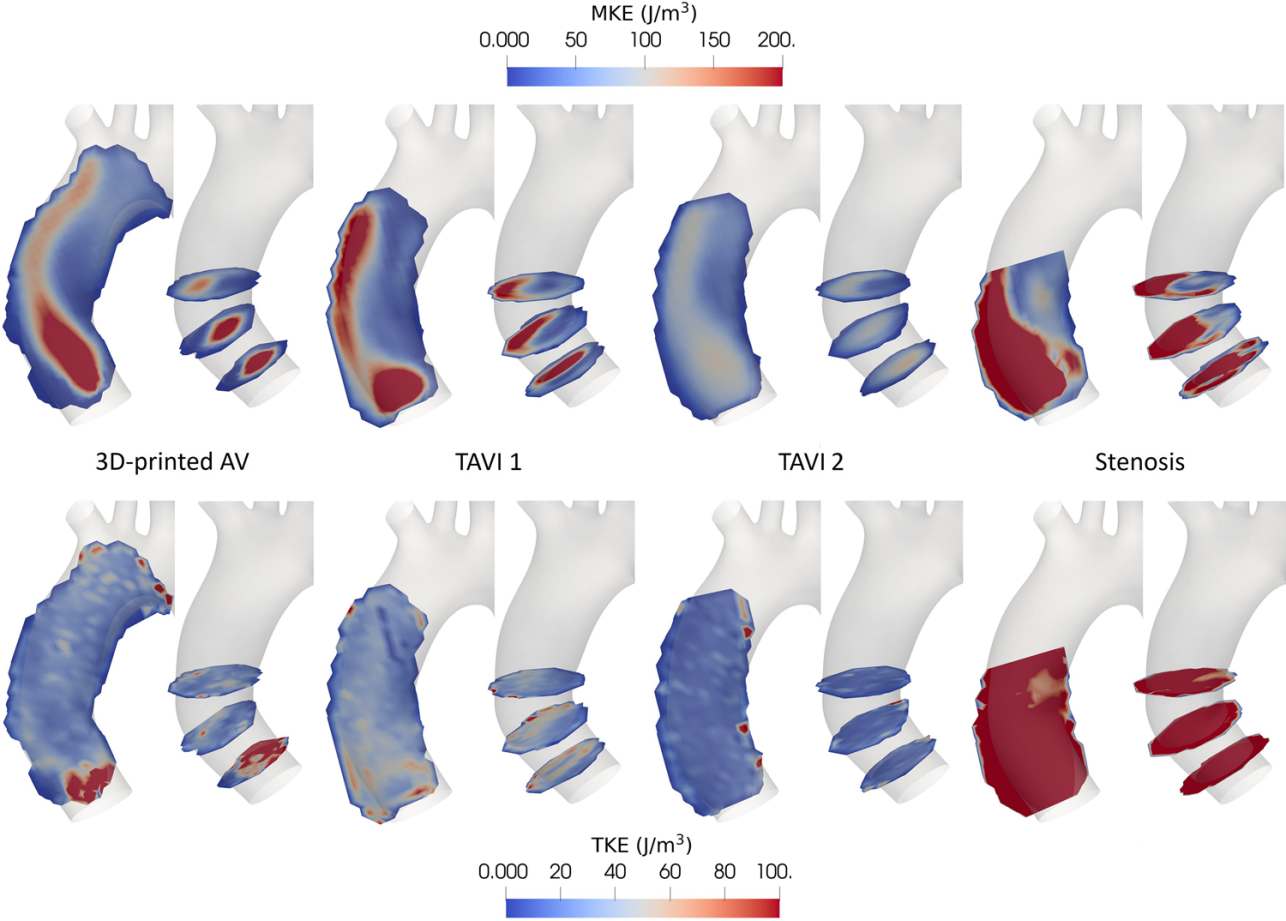
MKE (top) and TKE (bottom) at peak systole in one coronal plane and three cross-sections for the four studied cases.

The spatial distribution of kinetic energies allows highlighting areas of elevated flow velocities or higher shear as well as regions where flow might be disturbed. Figure 3 illustrates the spatial distribution of mean kinetic energy (MKE) and turbulent kinetic energy (TKE) at peak systole for all four cases. In general, we note higher MKE and TKE in the stenotic valve model compared to the other cases throughout the domain, as well as localized patches of elevated TKE for the 3D-printed AV.

To quantify the performance of the 3D-printed aortic valve, we investigate spatial averages of the kinetic energies, helicity intensity and viscous dissipation rates, presented in Table 1, as well as regurgitation. The ratio of retrograde flow volume through the valve to the stroke volume, called regurgitation, is a critical parameter when assessing the performance of synthetic aortic valves. As we cannot measure regurgitant flow through the valves directly, it was approximated by the time-integrated retrograde flux through a cross-section close to the valve. In our in vitro measurements, regurgitation was measured to be $6.7\,\%$ for the 3D-printed AV, $4.5\,\%$ for TAVI 1 and $5.2\,\%$ for TAVI 2. In Table 1, we also present the results normalized by a Reynolds number defined based on the aorta diameter as a reference length scale, $Re_{ao}^2$, (italicized values) for better comparability, as cardiac output (CO) differs between the considered cases. This normalization is motivated by the observation that TKE scales with the square of $Re_{ao}$^24^, which is proportional to CO. Spatially averaged MKE and TKE over one pulse are shown in Figs. S6 and  S7 in the SM.

**Table 1:** Comparison of spatially averaged kinetic energies, dissipation rates and helicity intensity for the different cases. Italicized results are normalized with the respective $Re_{ao}^2$.

Parameter	Units	3D-printed AV	TAVI 1	TAVI 2	Stenosis
Pulse avg. MKE	$\textrm{J}/\textrm{m}^{3}$	23.76	53.88	20.40	164.04
	$\textit{10}^{\textit{-5}}{} \textit{J}/\textit{m}^\textit{3}$	$\textit{5.2}$	$\textit{7.4}$	$\textit{3.2}$	$\textit{12.3}$
Peak MKE	$\textrm{J}/\textrm{m}^{3}$	63.36	134.4	54.00	570.36
	$\textit{10}^{\textit{-5}}{} \textit{J}/\textit{m}^\textit{3}$	$\textit{13.9}$	$\textit{18.6}$	$\textit{8.4}$	$\textit{42.7}$
Pulse avg. TKE	$\textrm{J}/\textrm{m}^{3}$	24.00	16.08	9.12	99.48
	$\textit{10}^{\textit{-5}}{} \textit{J}/\textit{m}^\textit{3}$	$\textit{5.3}$	$\textit{2.2}$	$\textit{1.4}$	$\textit{7.5}$
Peak TKE	$\textrm{J}/\textrm{m}^{3}$	35.04	30.96	13.19	258.12
	$\textit{10}^{\textit{-5}}{} \textit{J}/\textit{m}^\textit{3}$	$\textit{7.7}$	$\textit{4.3}$	$\textit{2.1}$	$\textit{19.3}$
Pulse avg. $h_2$	(-)	3.85	5.74	1.93	13.44
Pulse avg. $\epsilon _{\textrm{m}}$	$\textrm{W}/\textrm{m}^{3}$	12.60	23.88	5.76	66.48
	$\textit{10}^{\textit{-5}}{} \textit{W}/\textit{m}^\textit{3}$	$\textit{2.8}$	$\textit{3.3}$	$\textit{0.9}$	$\textit{5.0}$
Peak $\epsilon _{\textrm{m}}$	$\textrm{W}/\textrm{m}^{\textrm{3}}$	32.40	56.16	15.01	291.36
	$\textit{10}^{\textit{-5}}{} \textit{W}/\textit{m}^\textit{3}$	$\textit{7.1}$	$\textit{7.8}$	$\textit{2.3}$	$\textit{21.8}$
Pulse avg. $\epsilon _{\textrm{t}}$	$\textrm{W}/\textrm{m}^{3}$	213.84	175.08	34.68	1684.80
	$\textit{10}^{\textit{-5}}{} \textit{W}/\textit{m}^\textit{3}$	$\textit{46.8}$	$\textit{24.2}$	$\textit{5.4}$	$\textit{126.3}$
Peak $\epsilon _{\textrm{t}}$	$\textrm{W}/\textrm{m}^{3}$	589.44	395.64	80.28	8290.32
	$\textit{10}^{\textit{-5}}{} \textit{W}/\textit{m}^\textit{3}$	$\textit{129.0}$	$\textit{54.6}$	$\textit{12.5}$	$\textit{621.3}$

## Discussion

Trans- and paravalvular leakage is a common complication accompanying valve replacements and directly affects cardiovascular efficiency. ISO-5840-3:2013 regulates minimum device performance requirements for artificial aortic valves and stipulates regurgitation may not exceed $20\,\%$ for a 21 mm valve. The measured regurgitation fractions for the 3D-printed AV and the two TAVIs are in good agreement with leakage fractions measured directly in a pulse duplicator in previous studies^12,22^. In terms of leakage, the 3D-printed AV performs similarly to both TAVI 1 and 2 and fulfills the ISO-norm requirements.

The flow patterns downstream of the 3D-printed AV show a jet impingement on the outer aortic wall and a subsequent formation of helical flow, similar to the flow patterns distal to TAVI 2. For TAVI 1 the flow patterns follow a similar trend though the rotational flow at peak systole forms closer to the valve and is predominantly right-handed. Systolic flow distal to the stenosis is rotational immediately downstream of the valve as well as considerably less organized throughout the domain.

The flow downstream of all three valve cases on average is characterized by counter-rotating helical structures as indicated by the isosurfaces in Fig. 2. This is completely lacking in the stenotic case. Rather, we see right-handed helical structures close to the stenosed valve and left-handed ones further downstream. Helical flow in general^14^ and also counter-rotating helical structures have been shown to be present in the AAo of healthy subjects^16,25,26^. Based on considerations of the basic role of helicity in organising flows and in suppressing instabilities, Morbiducci *et al.*^16^ argue coherent helical structures in healthy aortic flow lead to more efficient perfusion and attenuate turbulence.

The temporal evolution of helical structures is similar for the 3D-printed AV and TAVI 2. In essence, this same time sequence of the flow topology was found in *in vivo* assessments of healthy subjects^16,25^. Although the average flow topology downstream of TAVI 1 is similar, the temporal sequence differs from the other two valve cases. For TAVI 1, counter-rotating helical structures develop already during early systole compared to at the systolic peak. Thus, the valve design not only impacts the flow topology but also the chronological sequence different topology states are observed. The temporal evolution of the helical patterns for the stenosis differs significantly from the valve cases, especially during systole and the decelerating phase. Interestingly, left-handed helical structures characterize most of the diastolic phase in all cases.

The spatial distribution of MKE of both the 3D-printed AV and TAVI 1 shows the aortic jet clearly. The jet of the 3D-printed AV is more centered and symmetric compared to TAVI 1, where the jet impinges on the aortic wall earlier. Further, MKE remains higher further downstream for TAVI 1 than the 3D-printed AV. MKE distribution in the flow field of TAVI 2 is qualitatively similar to the 3D-printed AV, though at much reduced MKE levels. This is likely due to an earlier opening of TAVI 2 owing to its significantly thinner leaflets (see Table 2) leading to a less pronounced systolic jet. The jet downstream of the stenosis is less confined than for the valve cases and elevated MKE is found in a substantial part of the investigation domain. Regions of high MKE indicate elevated velocities and sharp MKE gradients evince increased shear.

TKE is relatively uniform and low in the flow distal to TAVI 2, whereas TKE levels for the 3D-printed AV and TAVI 1 are consistently slightly higher. For both those cases, regions of elevated TKE close to the valve indicate local flow disturbances caused by the valves. The 3D-printed AV appears to cause a stronger but more localized flow disturbance compared to TAVI 1. TKE is also increased towards the aortic branches for the 3D-printed AV due to the bifurcation of the flow. These patches of elevated TKE first appear at peak systolic flow and persist through early diastole. During systole and early diastole, moderately increased TKE levels in the vicinity of the aortic valve as well as close to the aortic branches have also been reported in healthy subjects^18^. TKE is high throughout the investigation volume downstream of the stenosis, highlighting the disturbance of the flow by the stenosis.

Both pulse averaged and peak MKE for the 3D-printed valve are in the range of the TAVIs, whereas for the stenosis are significantly larger. Similarly to MKE, the time averaged and peak laminar dissipation rates as well as the helicity intensity values are in the same range for all three valve cases though considerably larger for the stenosis. The similar dissipation rates of MKE evince viscous losses due to laminar flow are comparable for the 3D-printed AV and the two TAVI devices. The quantity $h_2$ entails the overall amount of helical flow throughout one cycle. In a study covering 12 healthy and 16 diseased subjects, Lorenz *et al.*^26^ found significantly higher average values of helicity density in diseased patients’ aortas. Numerical studies of patient-specific aorta geometries reported $h_2$ values of $3.3 - 4.1$ for healthy subjects and $3.6 - 10.3$ for geometries with aneurysms^19,27^. This indicates a correlation between enhanced helicity intensity and various cardiovascular diseases. It is worth noting again though that helical flow is also a feature of normal healthy aortic flow albeit lower in magnitude. Our results show a good agreement where both TAVI cases exhibit significantly smaller $h_2$ than the stenotic case and are of the same order of magnitude as found in healthy aorta geometries. The helicity intensity of the 3D-printed AV lies inbetween the two TAVI cases, thus suggesting a helical flow amount comparable to healthy conditions.

Pulse averaged TKE for the stenotic case is about a factor 4 larger than any of the valve cases. The difference between the stenotic and the three valve cases is even more accentuated for peak TKE, which is about seven times higher than the 3D-printed AV. It should be noted that CO was considerably larger for the stenotic case than the valve cases. However, the difference remains significant when normalized by $Re_{ao}^2$, which accounts for the difference in forcing. Both pulse averaged and peak TKE normalized by $Re_{ao}^2$ are larger for the 3D-printed AV compared to the TAVIs. We can also take into account that the valves differ in their effective orifice areas and normalize our results by the square of the orifice Reynolds number, $Re_{vo}^2$, which is based on the equivalent orifice area diameter $D_{vo}$. This normalization allows for a comparison of valve performance accounting for variable forcing conditions and valve opening sizes. Nomalized by $Re_{vo}^2$, pulse averaged TKE is 2.5 times larger for the 3D-printed AV than TAVI1, while the ratio is roughly 1.9 for peak TKE. This might indicate that the 3D-printed AV is more prone to introduce turbulence than the TAVIs, possibly due to its overall geometry, surface texture or leaflet movements. Overall, it suggests the 3D-printed AV introduces more elevated flow disturbances than the TAVI valves though not nearly as much as a stenosed valve would. Ha *et al.*^18^ investigated turbulence levels in the aortic blood flow of a group of healthy test persons. Comparison of median TKE at peak systole normalized by $Re_{ao}^2$ reveals similar levels for all three valve cases ($2.1 - 6.4 \cdot 10^{-5} \textrm{J}/\textrm{m}^{3}$) as reported for healthy test persons ($6.1 - 8.1 \cdot 10^{-5} \textrm{J}/\textrm{m}^{3}$). Thus, the flow disturbances caused by the 3D-printed AV (as well as the TAVIs) are of a similar level as for native aortic valves. Median TKE for the stenosis, however, is one order of magnitude higher than the one reported for healthy test persons. Furthermore, we may also compare spatially averaged TKE both at peak systole and averaged over a pulse to other TAVI valves investigated in literature. Giese *et al.*^28^ studied the hemodynamics of five commercially available TAVI devices. Correcting for the extent of the integration volume, the spatially and pulse averaged TKE results reported by Giese *et al.* range from 0.7 to $1.8 \cdot 10^{-5} \textrm{J}/\textrm{m}^{3}$ when normalized by $Re_{ao}^2$ and from 1.6 to $3.7 \cdot 10^{-5} \textrm{J}/\textrm{m}^{3}$ when normalized by $Re_{vo}^2$. The results for all three valve cases presented here agree well with this range, with values of $0.6 \cdot 10^{-5} \textrm{J}/\textrm{m}^{3}$ and $3.5 \cdot 10^{-5} \textrm{J}/\textrm{m}^{3}$ for the 3D-printed AV, $0.2 \cdot 10^{-5} \textrm{J}/\textrm{m}^{3}$ and $1.3 \cdot 10^{-5} \textrm{J}/\textrm{m}^{3}$ for TAVI 1 and $0.1 \cdot 10^{-5} \textrm{J}/\textrm{m}^{3}$ and $0.8 \cdot 10^{-5} \textrm{J}/\textrm{m}^{3}$ for TAVI 2. Pietrasanta *et al.*^29^ investigated the influence of the valve position on blood flow and reported spatially averaged TKE of $0.3 - 0.6 \cdot 10^{-5} \textrm{J}/\textrm{m}^{3}$ (normalized by $Re_{ao}^2$) or $3.0 - 8.2 \cdot 10^{-5} \textrm{J}/\textrm{m}^{3}$ (normalized by $Re_{vo}^2$) at peak systole depending on valve location. Again, these values are in the range of the results of the 3D-printed AV ($0.9 \cdot 10^{-5} \textrm{J}/\textrm{m}^{3}$ and $5.1 \cdot 10^{-5} \textrm{J}/\textrm{m}^{3}$) and TAVI 1 ($0.3 \cdot 10^{-5} \textrm{J}/\textrm{m}^{3}$ and $2.7 \cdot 10^{-5} \textrm{J}/\textrm{m}^{3}$) whereas values are smaller for TAVI 2 ($0.2 \cdot 10^{-5} \textrm{J}/\textrm{m}^{3}$ and $1.2 \cdot 10^{-5} \textrm{J}/\textrm{m}^{3}$).

In terms of pulse averaged turbulent dissipation rate, the stenosis differs strongly from the valve cases. Although the peak turbulent dissipation rate of the 3D-printed AV is almost $50\,\%$ larger than for TAVI 1, it is still in the same order of magnitude. Peak $\epsilon _{\textrm{t}}$ for the stenotic case, however, is one order of magnitude larger than for the 3D-printed AV even after correcting for the different CO. Thus, viscous losses due to turbulent dissipation are significantly lower in the flow downstream of the 3D-printed AV than following the severe aortic stenosis studied here. Considering both laminar and turbulent dissipation, total irreversible energy losses for the 3D-printed AV are considerably lower than for a stenosed case, though still higher than for the two TAVIs. Irreversible energy losses negatively impact the efficiency of the cardiovascular system and cause an increased cardiac afterload, i.e. increased contracting effort of the left ventricle. Consequently, the 3D-printed aortic valve is less efficient than the two TAVI devices, however it still constitutes a significant improvement over a disease like aortic stenosis.

## Conclusion

We presented an assessment of the hemodynamics of a newly developped 3D-printed aortic valve. To the best of our knowledge, this study is the first of its kind to characterize blood flow through a 3D-printed and fully customizable aortic valve. Comparisons of various metrics to a bioprosthetic TAVI and a polymeric TAVI valve as well as to a severe aortic stenosis were made to classify the performance of the 3D-printed AV. Our analysis showed that the 3D-printed AV introduces healthy flow topology and patterns in the AAo similarly to the TAVI valves. Furthermore, it leads to a significant reduction of TKE levels as well as irreversible energy losses compared to the stenotic valve. Turbulence metrics are comparable with reported values for conventional TAVI devices and even healthy subjects, even though the performance of TAVI 1 and 2 could not be matched yet. Together with the fast and inexpensive manufacturing and the promise of patient-specific valve geometries, these results demonstrate the 3D-printed silicone valve’s suitability as realistic aortic valve phantoms and underpin the future potential of 3D-printed AVs as fully customizable prosthetic aortic heart valves. We anticipate that the present work may contribute in guiding further development of fully customizable aortic valve prostheses.

## Limitations

The aorta model used in this study features the AAo, the AoA and branches as well as part of the DAo. The sinuses of Valsalva are neglected. Further, only one aorta geometry was studied. For this study, we investigated one position and orientation for each of the valves and stenosis model. Variations of valve position and orientation may influence the blood flow. Furthermore, the blood analogue fluid is Newtonian, whereas real blood is a shear-thinning fluid. The CO imposed by the VAD and the pneumatic pump is approximately $3 \,\mathrm {l/min}$ for the valve cases, which is relatively low compared to typical, healthy in vivo conditions. Due to the limited performance of the pneumatic pump the systolic period was set to $50\,\%$ of the pulse period. This was to ensure a full displacement of the fluid volume provided by the VAD. While we expect that the qualitative trends in terms of valve performance remain the same at stronger forcing conditions, it would be important to extend the comparison to high cardiac outputs and peak flow values. This is however out of the scope of the present paper, as it would require a different setup (e.g. a mechanical piston pump as used in Coulter *et al.*^12^). Our results are based on an in vitro system that mimics many aortic flow features realistically, including a patient specific aorta geometry, compliant wall boundaries and realistic waveform shapes, while compromising on the peak magnitudes. While the latter are small compared to typical in vivo conditions, the findings are still relevant for hemodynamics in real aortas.

## Methods

**Figure 4:**
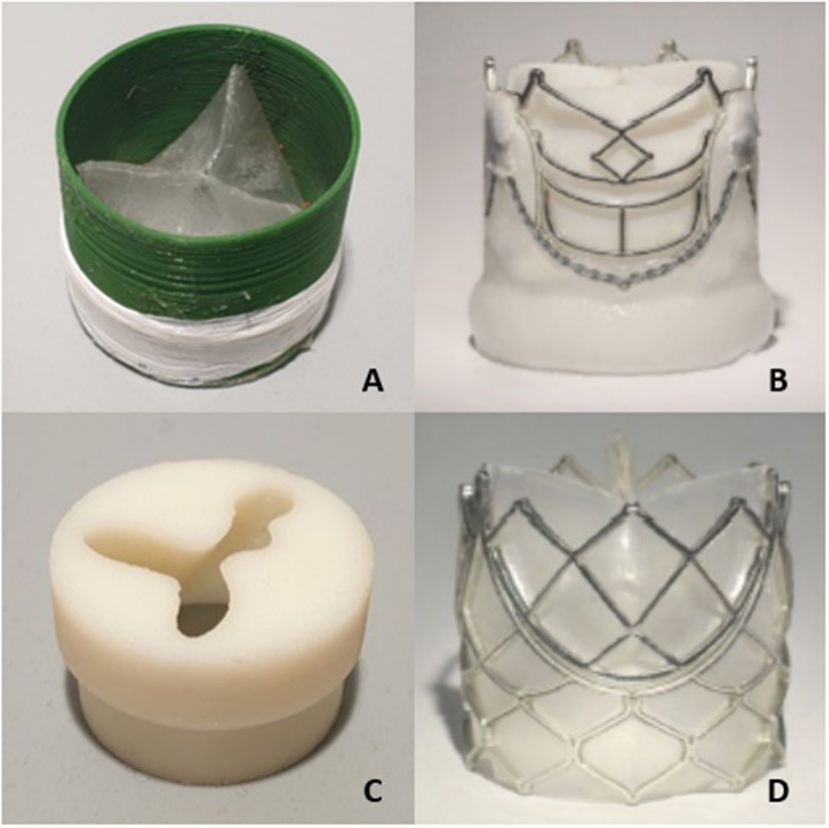
The aortic valves investigated in this study. **(A)** 3D-printed AV, **(B)** TAVI 1 (SAT, B-TAVR), **(C)** severe stenosis and **(D)** TAVI 2 (SAT, P-TAVR).

The blood flow downstream of a 3D-printed aortic valve (3D-printed AV) is studied in vitro and compared to a severe aortic stenosis model and two TAVI devices, which were previously investigated in separate studies^22,24^. The different valves are shown in Fig. 4. The two TAVI valves are both balloon-expandable valves with a size of 23 mm developed by Strait Access Technologies (SAT), South Africa^30^. The specific models are the B-TAVR and the P-TAVR products, herein called TAVI 1 and TAVI 2, respectively. TAVI 1 is a tissue valve with decellularized bovine pericardial leaflets sealed with a polymer skirt. TAVI 2 features polymeric leaflets and sealing, as well as stent scaffolds designed to minimize the stresses in the stent and leaflets. Leaflets of TAVI 1 are thicker than for TAVI 2 and the two devices further differ in their natural shape, where TAVI 1 is naturally neutral and TAVI 2 is closed. The two TAVI models are being developed with commercial intent and first-in-man clinical trials are ongoing at the time of writing. The stenosis model is a rigid 3D-print emulating the geometry of a tricuspid aortic valve stenosed due to leaflet calcification with an orifice area of $0.75\,\textrm{cm}^2$.

### 3D-printed AV manufacturing

The 3D-printed AV features a bio-inspired tri-leaflet geometry which can be easily customized. Due to the significant impact of effective orifice area (EOA) of a valve on pressure loss and turbulence in the aortic flow, we chose to match the effective orifice area of the 3D-printed AV to the TAVIs (see Table 2). This results in a size of $21\,\textrm{mm}$ for the 3D-printed AV, which is a smaller size than the TAVIs, whereas a 3D-printed AV of the same size would exhibit a significantly larger EOA. The valve was produced using the manufacturing process presented by Coulter *et al.*^12^. A biocompatible silicone resin is sprayed onto a mandrel of the aortic valve geometry, creating the three leaflets. This process ensures good shape accuracy and uniform thickness of the leaflets. The silicone valve is then mounted onto a cylindrical support structure 3D-printed in polylactide, allowing for an easy insertion into the aorta model. The technology also allows for the inclusion of a silicone membrane matching the aortic root, derived from patient computer tomography data, to ensure the best fit in real geometries. The additive silicone manufacturing is both time-efficient and inexpensive, especially compared to conventional tissue valve manufacturing. During initial testing of the 3D-printed AV under accelerated conditions^12^, it showed excellent durability as well as opening and closing behavior.

### Experimental setup

**Figure 5:**
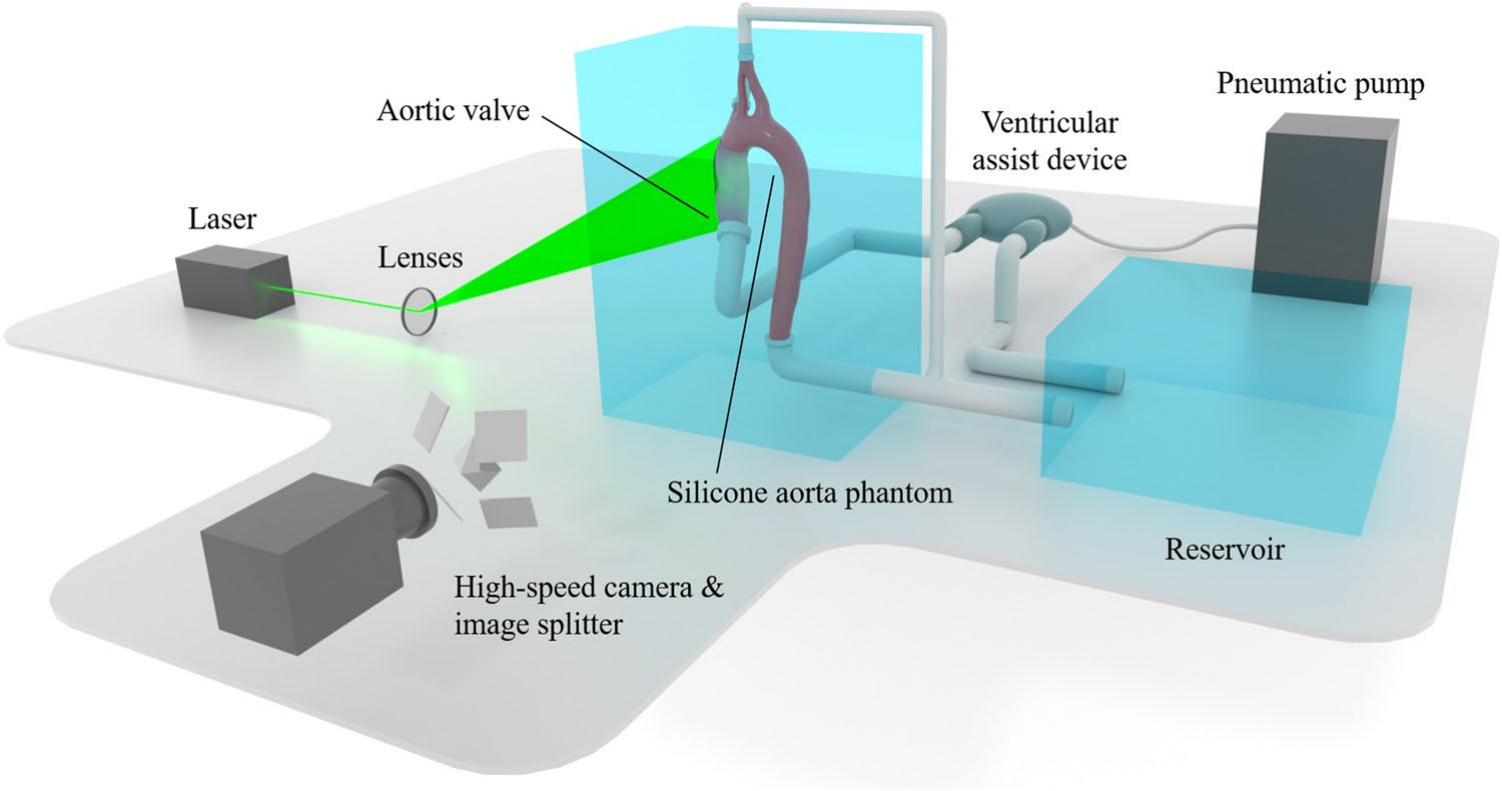
Schematic illustration of the experimental setup.

The 3D-PTV setup consists of a mock circuit and an optical component (see Fig. 5). The mock circuit comprises a 80 ml ventricular assist device (VAD, MEDOS, Germany), a pneumatic pump (Berlin Heart, Germany) and an anatomically accurate silicone aorta replica (Elastrat Sarl, Switzerland). The VAD is accentuated by a waveform pressure imposed by the pneumatic pump, resulting in inflow conditions close to physiological ones in the silicone aorta (see Figs. S2–S4 in the SM). The aorta replica geometry has been obtained from a magnetic resonance scan of a healthy patient, has an inlet diameter of $d_{ao} = 21\,\textrm{mm}$ and features the ascending aorta (AAo), the aortic arch (AoA) including branches and part of the descending aorta (DAo). The straight inflow section upstream of the valve position is approximately 20 cm ($\sim 9d_{ao}$) long, which is enough to eliminate the majority of the flow profile’s skewness^31^.

All three valve devices and the stenosis model were inserted into the aorta phantom at the same location at the inlet. Special attention was paid to the consistent orientation of the valves and the stenosis by aligning one leaflet commissure to a mark on the aorta replica at the outer curvature. The size of the TAVIs is slightly larger than the aorta inlet, which is a common practice in aortic valve replacements to securely implant the device. The size of the 3D-printed AV was chosen to be equal to the aorta inlet diameter ensuring a similar effective orifice area for all three valve cases (see Table 2).

The blood analogue fluid is a mixture of water ($48\,\textrm{m}\%$), glycerine ($37\,\textrm{m}\%$) and sodium chloride ($15\,\textrm{m}\%$)^32^. This mixture is a Newtonian fluid with a kinematic viscosity of ${\nu = 4.85\cdot 10^{-6}\,\mathrm {m^2/s}}$, a density of ${\rho = 1200\,\mathrm {kg/m^3}}$ and a refractive index of $n = 1.41$ matching that of the silicone used in the aorta phantom. To reduce any optical refractions, the aorta phantom is contained in a box filled with the same liquid. The tracer particles are neutrally buoyant, fluorescent rhodamine-dyed particles with a diameter between 180 and $250\,\mu \textrm{m}$^15^. The corresponding Stokes number is approximately 0.01, which means that the tracer particles can be expected to follow the streamlines faithfully.

The optical part of the setup mainly consists of a high-speed camera and a laser. The camera (Fastcam SA5, Photron, Japan) has a full resolution of $1024\,\times \,1024$ pixels at a maximal frame rate of 7000 frames per second. A Nikon AF Micro Nikkor $60\,\textrm{mm}$ $\textrm{f}/2.8$ D lens (Nikon, Japan) was used in combination with an orange bandpass filter. An array of carefully positioned mirrors split the recorded image in four views with different orientations. A diode-pumped Nd-YLF laser (Quantronix, Darwin Duo $527\,\textrm{nm}$, USA), a beam expander and spherical lenses were used to illuminate the observation volume.

### Measurement procedure

**Figure 6:**
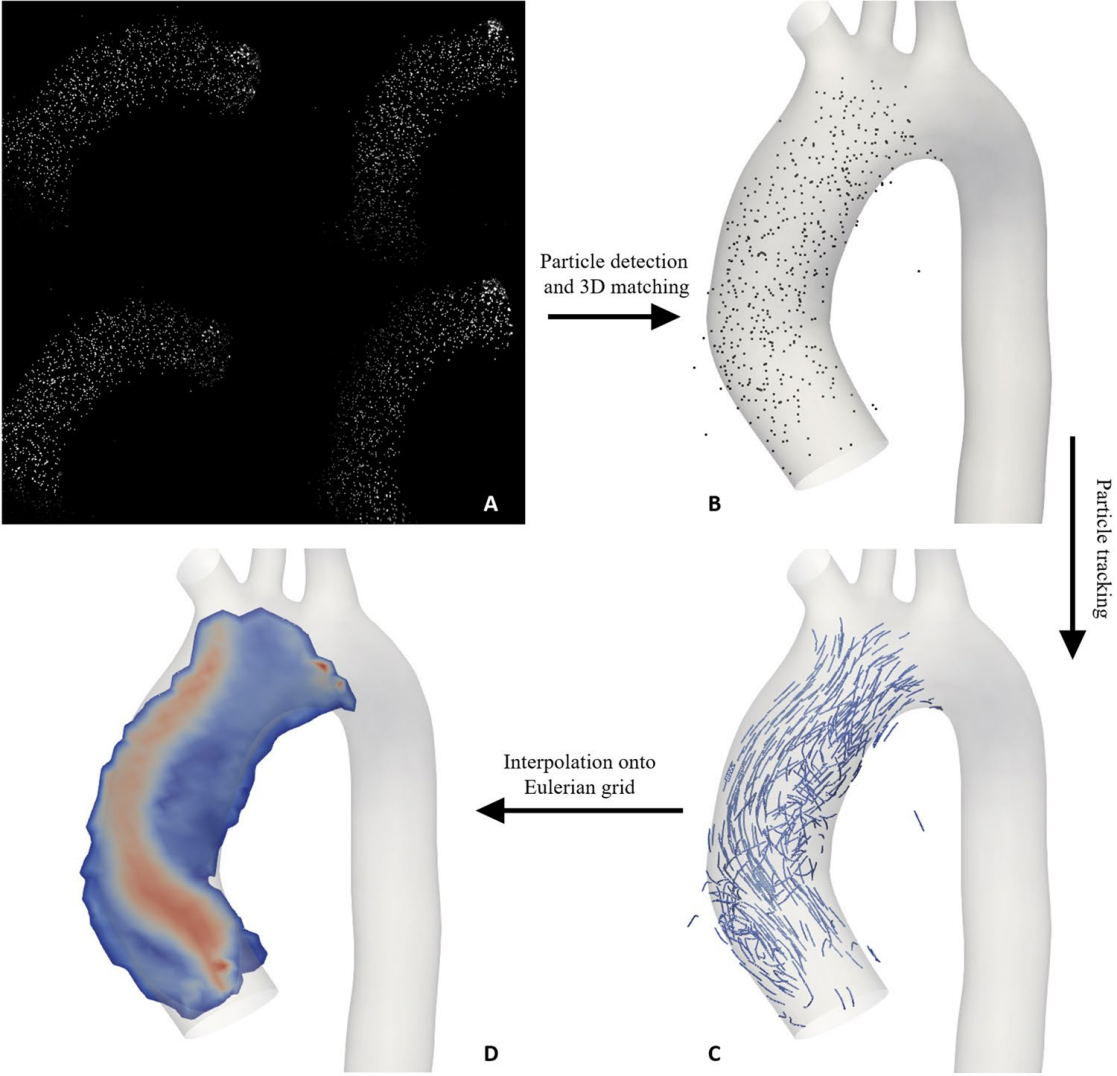
Different steps in 3D-PTV. From the raw quartered image **(A)** particles are detected and matched between the four views to obtain the three-dimensional particle positions **(B)**. Tracking of the particles between subsequent frames yields Lagrangian particle trajectories **(C)**. Eulerian flow fields **(D)** are obtained using a grid interpolation.

3D-PTV is an optical measurement technique capable of capturing three-dimensional instantaneous flow velocities by tracking tracer particles from four views of different orientation^33^. Particle velocities are calculated along the Lagrangian trajectories, resulting in a 3D point cloud of velocity data at each time instant. Calibration, particle detection and matching between the different orientations as well as the particle tracking were carried out using the open source software OpenPTV (https://www.openptv.net/)^33,34^. For all investigated cases, 30 cycles were recorded at a frame rate of $2000\,\textrm{Hz}$ ($7000\,\textrm{Hz}$ for the stenosis case), resulting in $50'000$ ($175'000$) frames in total (Fig. S1 in the SM shows the convergence of TKE as a function of the number of repetitions). Instantaneous Eulerian flow fields were then computed by interpolating the point cloud velocity data on a grid with a voxel size of $2.5\,\textrm{mm}\,\times \,2.5\,\textrm{mm}\,\times \,2.5\,\textrm{mm}\,$. To increase the point density necessary for a sufficiently high spatial resolution of the Eulerian grid, 60 consecutive time instants were combined per interpolation step, resulting in a final coarse-grained temporal resolution of 33 Hz (116 Hz for the stenosis case). The resulting voxel-wise velocity uncertainty was ca. $4.2\cdot 10^{-4}\,\mathrm {m/s}$  ^15^. Figure 6 illustrates the different steps of a 3D-PTV measurement and post-processing. The camera was synchronised to the pump, ensuring the beginning of a recording always corresponds to the start of a new cycle. The flow conditions defined by the pneumatic pump and the VAD were within physiological range for all cases and are summarized in Table 2. Here, the Reynolds number is $Re = 4Q/(d\pi \nu )$ and the Womersley number is $Wo = d_{vo}/2(2\pi f/\nu )^{0.5}$ with *Q* being the volumetric flux, *d* the diameter and *f* the pulse frequency. Mean Reynolds numbers are defined with the mean volumentric flux and peak Reynolds numbers with the volumetric flux at peak systole. $Re_{ao}$ is based on the aortic diameter $d_{ao}$, whereas $Re_{vo}$ is based on the equivalent effective valve orifice area diameter $d_{vo}$. CO varied between cases with the relative standard deviation being $23\,\%$ considering all cases, though less than $12\,\%$ for the three valve cases. The larger difference of the stenosis case can be attributed to the use of a slightly bigger VAD for this case. The differences between valve cases are likely caused by small variations in the experimental boundary conditions, namely height of the reservoir, length in tubing and pump inaccuracies.

**Table 2:** Experimental conditions for all four investigated cases.

Parameter	3D-printed AV	TAVI 1	TAVI 2	Stenosis
Mean volumetric flux (mL/s)	51.49	64.85	61.02	87.66
Pulse frequency (Hz)	0.83	0.90	0.90	0.87
Cardiac output (L/min)	3.09	3.89	3.66	5.28
Stroke volume (mL)	61.80	72.06	67.86	101.29
Mean $Re_{ao}$ (-)	644	811	763	1100
Peak $Re_{ao}$ (-)	1327	1243	1147	3225
Mean $Re_{vo}$ (-)	825	1064	1055	2364
Peak $Re_{vo}$ (-)	1700	1632	1587	6931
Wo (-)	10.91	12.42	12.42	5.19
Effective orifice area ($\textrm{cm}^2$) $^*$	2.11	2.01	1.81	0.75
Leaflet thickness ($\mu \textrm{m}$)	300	602	152	-

$^*$ Effective orifice areas were determined in a pulse duplicator for a CO of 5 L/min in previous studies (3D-printed AV^12^; TAVI 1 and 2^22^).

### Clinically relevant Eulerian parameters

Elevated turbulence levels and disturbed flow fields are commonly correlated with various cardiovascular diseases^7^. Consequently, several fluid dynamic quantities, which have been previously shown to have physiological relevance in the context of aortic blood flows have been used to characterize hemodynamics and disturbed blood flow in particular^7,23^. Below, we introduce the metrics used in this study to assess the flow downstream of the valves both qualitatively and quantitatively. The basis for the computation of these quantities are the Eulerian velocity fields. Reynolds decomposition is applied to the instantaneous velocity to separate the mean velocity field $\overline{\textbf{u}}$ from the velocity fluctuations $\textbf{u}^\prime$, due to turbulent fluctuations. Thus,

1 $$\begin{aligned} \textbf{u}_{\textrm{inst}} = \overline{\textbf{u}} + \textbf{u}^\prime , \end{aligned}$$

where vector quantities are written in bold. The mean velocity is computed by applying a phase-averaging over all recorded cycles as the forcing is periodic,

2 $$\begin{aligned} \overline{\textbf{u}}(\textbf{x},t) = \frac{1}{n}\displaystyle \sum _{i=1}^{n}\textbf{u}_{\textrm{inst}}(\textbf{x},t + iT), \end{aligned}$$

where *n* is the number of cycles and *T* is the period of the cycle. Similarly, the energy contained in the flow can be decomposed into the mean and the turbulent kinetic energy. The mean kinetic energy (MKE) is associated with the mean velocity field whereas the turbulent kinetic energy (TKE) is carried by the fluctuating field. MKE and TKE are defined as,

3 $$\begin{aligned} \textrm{MKE}= & {} \rho \frac{1}{2}\displaystyle \sum _{i=1}^{3}{\overline{u}_i}^2, \end{aligned}$$

4 $$\begin{aligned} \textrm{TKE}= & {} \rho \frac{1}{2}\displaystyle \sum _{i=1}^{3}{u_i^\prime }^2, \end{aligned}$$

where $u^\prime _i$ and $\overline{u}_i$ are the components of the fluctuating and the mean velocity vector, respectively. Assessment of the kinetic energies yields information on turbulence levels in the flow and valve efficiency.

In turbulent flows, energy is cascaded from larger to smaller eddies and ultimately dissipated into heat at the smallest scales^35^. The total viscous dissipation of kinetic energy is given by,

5 $$\begin{aligned} \epsilon = \epsilon _m + \epsilon _t, \end{aligned}$$

the sum of the viscous dissipation of MKE, $\epsilon _m$, and that of TKE, $\epsilon _t$. The dissipation of MKE is defined as,

6 $$\begin{aligned} \epsilon _m = 2\nu \overline{S}_{ij}\overline{S}_{ij}, \end{aligned}$$

with the mean strain rate tensor $\overline{S}_{ij} = \frac{1}{2}(\frac{\delta \overline{u}_i}{\delta x_j}+\frac{\delta \overline{u}_j}{\delta x_i})$ and using Einstein notation. Similarly, the dissipation of TKE is defined as,

7 $$\begin{aligned} \epsilon _t = 2\nu \overline{s^\prime _{ij}s^\prime _{ij}}, \end{aligned}$$

where $s^\prime _{ij} = \frac{1}{2}(\frac{\delta {u^\prime }_i}{\delta x_j}+\frac{\delta {u^\prime }_j}{\delta x_i})$ is the fluctuating strain rate tensor. While $\epsilon _m$ can be directly computed from the mean flow field, assessing $\epsilon _t$ requires the spatial resolution of the instantaneous velocity to be on the order of the Kolmogorov length scale, the smallest length scale of turbulence. Such highly resolved data is challenging to measure and cannot be achieved with the 3D-PTV setup for this study, since it focuses on an observation volume extending over a substantial part of the AAo. Hence, we use as an approximation of $\epsilon _t$ the shear scaling method proposed by Gülan *et al.*^24^, which was applied to aortic blood flows with satisfactory results. The estimated turbulent dissipation $\epsilon _t^{shear}$ can be computed as,

8 $$\begin{aligned} \epsilon _t^{shear} = c_\epsilon ^{shear} \Vert \overline{S}_{ij}\Vert \overline{u_i^\prime u_i^\prime }, \end{aligned}$$

where $\Vert \overline{S}_{ij} \Vert$ denotes the Frobenius norm of the mean rate-of-strain tensor and $c_\epsilon ^{shear}$ is the shear parameter which is approximately 0.1^36^. The two dissipation rates $\epsilon _{\textrm{m}}$ and $\epsilon _{\textrm{t}}$ may also be coined *laminar* and *turbulent* dissipation rate, respectively, due to the associated flow states. The dissipation rates quantify irreversible energy losses due to viscous dissipation.

Complex flows inherently exhibit rotation^35^, hence vorticity $\varvec{\omega }$, defined as the curl of the velocity vector,

9 $$\begin{aligned} \overline{\varvec{\omega }} = \nabla \times \overline{\textbf{u}}, \end{aligned}$$

is non-zero. Helicity describes a fluid’s potential to form helical flow patterns and is defined as the integration of the helicity density $H_k$, which is the inner product of the velocity and the vorticity vectors^37^, thus, the helicity density of the mean flow is,

10 $$\begin{aligned} \overline{H}_k = \overline{\textbf{u}} \cdot \overline{\varvec{\omega }}. \end{aligned}$$

The sign of the value of $H_k$ indicates the direction of rotation where a positive value corresponds to a right-handed and a negative value to a left-handed rotation. The spatial and temporal average of the helicity density is termed *helicity intensity* $h_2$, which indicates the total amount of helical flow^38^. It is defined as,

11 $$\begin{aligned} h_2 = \frac{1}{TV}\int _T\int _V \mid \overline{H}_k\mid \,\textrm{d}V\textrm{d}t, \end{aligned}$$

with *V* the investigation volume. It is obvious from its definition $h_2$ does not distinguish the direction of the helical flow. A common way to visualize the direction of helical structures is by computing the local normalized helicity (LNH) proposed by Shtilman *et al.*^39^. LNH of the time averaged flow is defined as,

12 $$\begin{aligned} \textrm{LNH}(\textbf{x},t) = \frac{\overline{\textbf{u}}(\textbf{x},t)\cdot \overline{\varvec{\omega }}(\textbf{x},t)}{\mid \overline{\textbf{u}}(\textbf{x},t)\mid \,\mid \overline{\varvec{\omega }}(\textbf{x},t)\mid }. \end{aligned}$$

LNH ranges between $-1$ and 1 and corresponds to the value of the local cosine between the velocity and vorticity vectors. Thus, the sign indicates the direction of the rotation analogously to $H_k$ and the magnitude is a measure for the helical degree of the flow, where values of 1 and $-1$ show purely helical flow and 0 suggests reflectional symmetric flow.

The investigation domain of the stenosis case extends less far downstream in the AAo than for the other three cases to achieve sufficient spatial resolution for this more complex flow case. Thus, spatial averages of any metrics were restricted to the investigation domain of the stenosis case to allow for a meaningful comparison of these statistics.

### Supplementary Information

Supplemental Information.

## Data Availability

The datasets generated and analysed during the current study are available in the ETH Research Collection: https://doi.org/10.3929/ethz-b-000559354.
