# Supplementary material for: In vitro investigation of the blood flow downstream of a 3D-printed aortic valve
Source: Sci Rep. 2024 Jan 18;14:1572. doi: 10.1038/s41598-024-51676-6 (PMC10796383; doi:10.1038/s41598-024-51676-6)
Supplement: Supplementary file 1 — Supplemental Information. [file 41598_2024_51676_MOESM1_ESM.pdf]

In Vitro Investigation of the Blood Flow Downstream of a 3D-printed Aortic Valve

Supplementary Material

Till Zeugin[1,2*], Fergal B. Coulter[3], Utku Gülan[4], André R. Studart[3], and Markus Holzner[2,5]

[1]Institute of Environmental Engineering, Dept. of Civil, Environmental and Geomatic Engineering, Swiss Federal Institute of Technology ETH Zürich, Zürich, Switzerland
[2]Swiss Federal Institute for Forest, Snow and Landscape Research WSL, Birmensdorf, Switzerland
[3]Complex Materials, Swiss Federal Institute of Technology ETH Zürich, Zürich, Switzerland
[4]Hi-D Imaging, Winterthur, Switzerland
[5]Swiss Federal Institute for Water Science and Technology EAWAG, Dübendorf, Switzerland
[*]zeugin@ifu.baug.ethz.ch

This document contains supporting information to the study *In Vitro Investigation of the Blood Flow Downstream of a 3D-printed Aortic Valve* mostly in the form of figures:

- Additional information on the 3D-PTV method
- Experimental forcing conditions
- Additional results

3D-PTV

In a previous study, our group cross-validated mean velocity and TKE evaluation using 3D-PTV and 4D-MRI in Knobloch *et al.*[1]. The two methods are based on entirely different acquisition times and data processing to obtain TKE, i.e. 32 cycles and estimation based on time-resolved Lagrangian velocity for 3D-PTV; $13-76$ min acquisition time and Bayesian multipoint MR velocity encoding. Satisfactory agreement was obtained with a relative error of better than 10 % for velocity and better than 15 % for TKE. We have also performed a convergence check for TKE of the present measurements. Figure S1 shows the normalized convergence of TKE for three regions in the observation volume. With 30 cycles considered, computed TKE differs from its converged value by 12 %, which is within the experimental uncertainty (ca. 15 %). Considering that every recorded pulse amounts to almost $10GB$ of data, there is a trade-off between improved accuracy and cost.

Experimental forcing conditions

Figure S2 shows the pressure applied to the ventricular assist device (VAD) by the pneumatic pump over one cycle, as well as a schematic of the functioning of the VAD. The systolic pressure is sufficient to completely compress the VAD's 80 mL chamber. Figure S3 shows the aortic pressure over one pulse measured for the 3D-printed AV case and Figure S4 the measured phase averaged net flow rate for the different cases.

Additional results

Figure S5 shows LNH isosurfaces for all investigated cases at four different instances over one pulse.

Figure S6 and S7 show the spatially averaged MKE and TKE, respectively, for all four inverstigated cases over one cycle.

[1]Knobloch, V., C. Binter, U. Gülan, A. Sigfridsson, M. Holzner, B. Lüthi, and S. Kozerke. Mapping mean and fluctuating velocities by bayesian multipoint mr velocity encoding-validation against 3d particle tracking velocimetry. Magn Reson Med, 71(4):1405-1415, 2014.

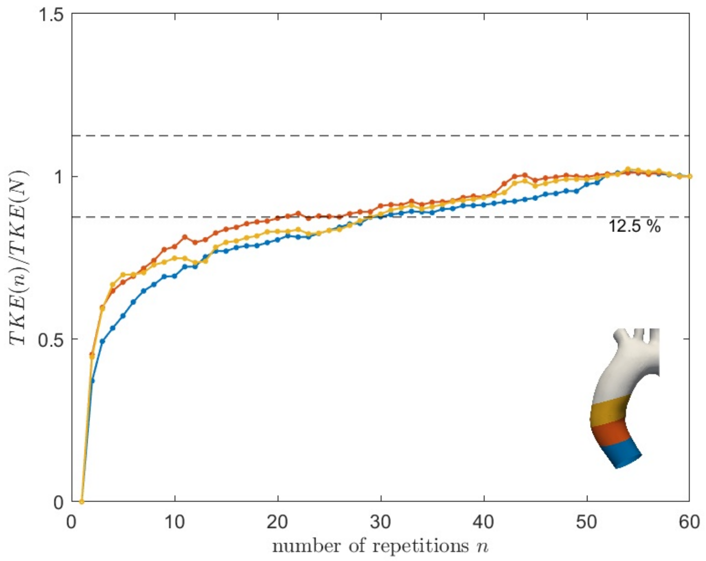

Figure S1. Normalized convergence of spatially averaged TKE at peak systole as a function of repetitions number n. The convergence is evaluated for three regions corresponding to the volumes defined by the aorta cross sections at distances 15 mm (blue), 30 mm (orange) and 45 mm (yellow) from the valve.

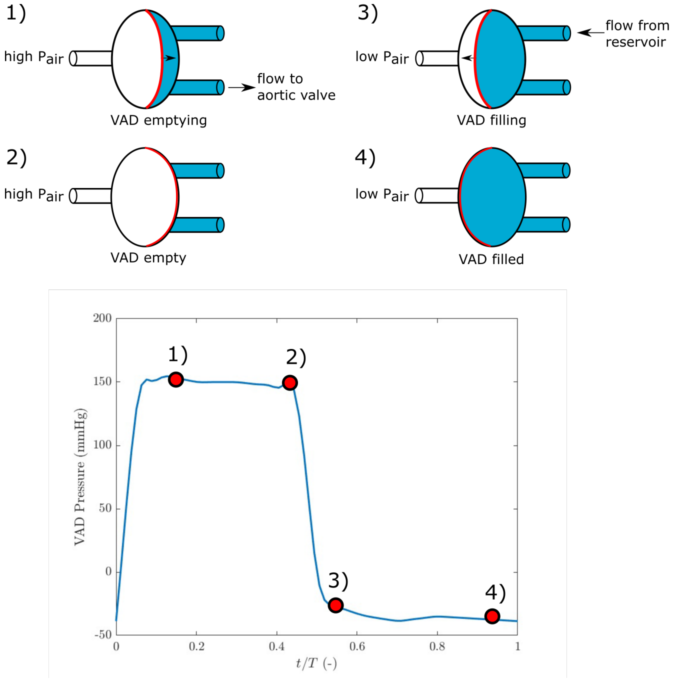

Figure S2. Pressure acting on the VAD over one cycle and schematic of the VAD functioning.

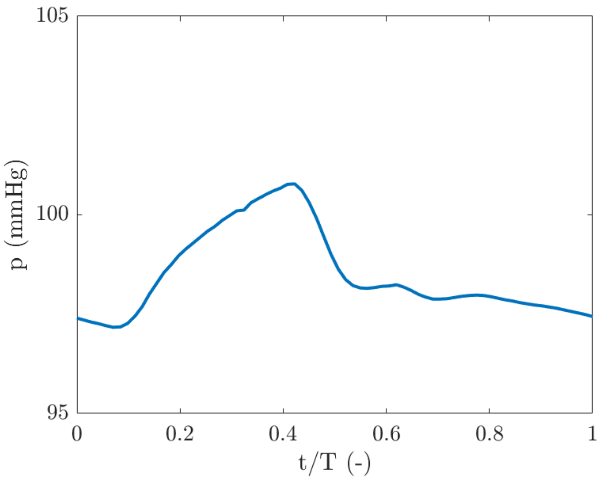

Figure S3. Pressure measured in the ascending aorta downstream of the 3D-printed AV over one cycle.

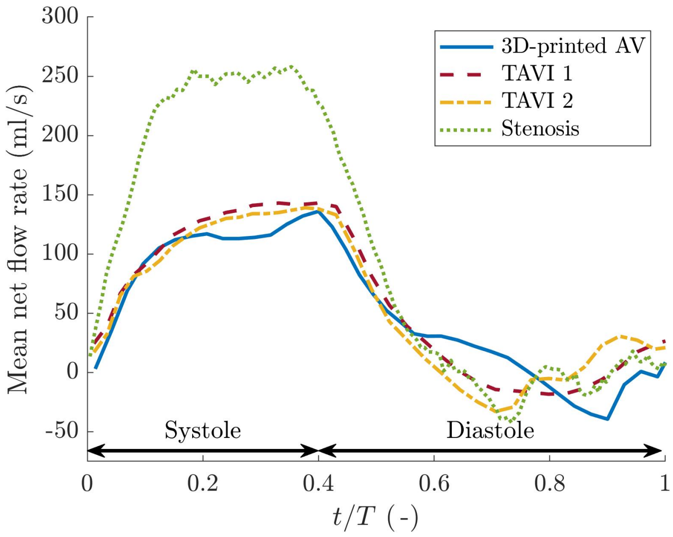

Figure S4. Phase averaged net flow rate for the different cases over one cycle.

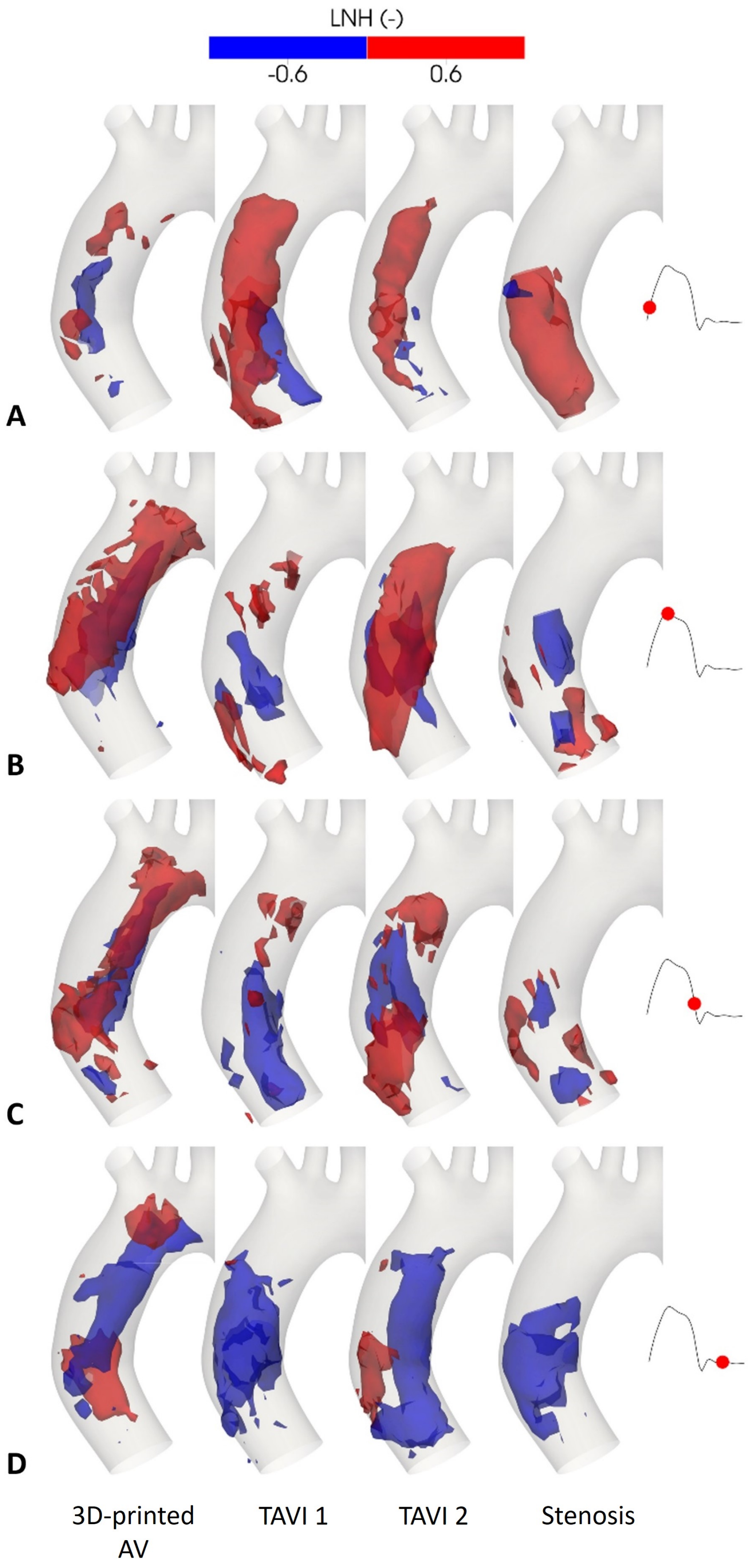

Figure S5. Local normalized helicity for the different cases at four instances over a pulse. **(A)** Accelerating phase, **(B)** peak systole, **(C)** decelerating phase and **(D)** diastole. Positive (negative) values indicate right-(left-)handed fluid structures.

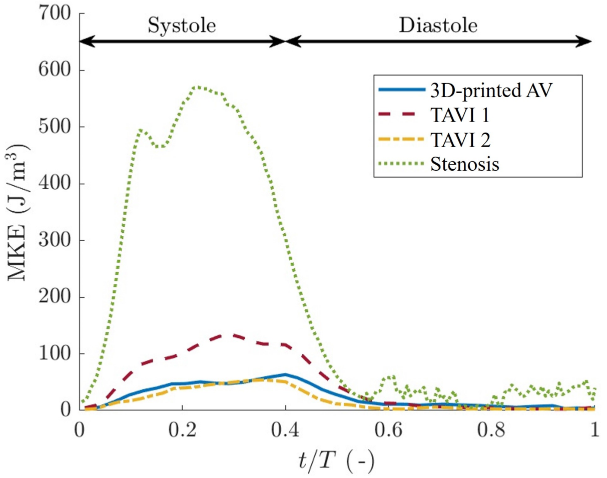

Figure S6. Spatially averaged MKE over one cycle.

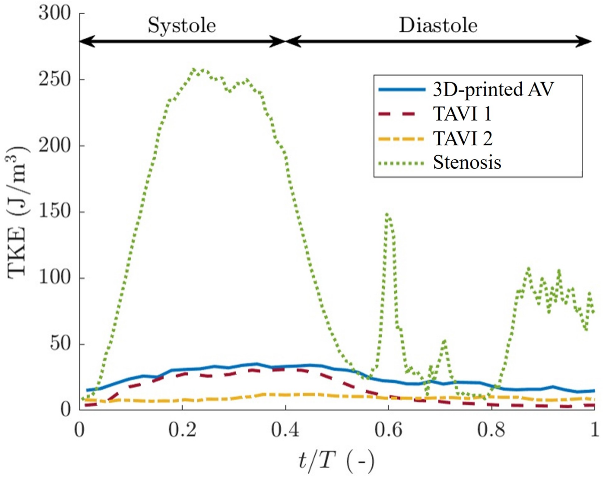

Figure S7. Spatially averaged TKE over one cycle.
